# Manure applications alter the abundance, community structure and assembly process of diazotrophs in an acidic Ultisol

**DOI:** 10.3389/fmicb.2022.965293

**Published:** 2022-08-12

**Authors:** Yongxin Lin, Guiping Ye, Hang-Wei Hu, Jianbo Fan, Ji-Zheng He

**Affiliations:** ^1^Key Laboratory for Humid Subtropical Eco-Geographical Processes of the Ministry of Education, School of Geographical Sciences, Fujian Normal University, Fuzhou, China; ^2^Institute of Oceanography, College of Geography and Oceanography, Minjiang University, Fuzhou, China; ^3^School of Agriculture and Food, Faculty of Veterinary and Agricultural Sciences, The University of Melbourne, Parkville, VIC, Australia; ^4^State Key Laboratory of Soil and Sustainable Agriculture, Institute of Soil Science, Chinese Academy of Sciences, Nanjing, China

**Keywords:** diazotroph, soil aggregates, manure application, assembly process, agroecosystem

## Abstract

The excessive usage of nitrogen (N) fertilizers can accelerate the tendency of global climate change. Biological N fixation by diazotrophs contributes substantially to N input and is a viable solution to sustainable agriculture *via* reducing inorganic N fertilization. However, how manure application influences the abundance, community structure and assembly process of diazotrophs in soil aggregates is not fully understood. Here, we investigated the effect of manure amendment on diazotrophic communities in soil aggregates of an arable soil. Manure application increased soil aggregation, crop yield and the abundance of *nifH* genes. The abundance of *nifH* genes increased with aggregate sizes, indicating that diazotrophs prefer to live in larger aggregates. The abundance of *nifH* genes in large macroaggregates, rather than in microaggregates and silt and clay, was positively associated with plant biomass and crop yield. Both manure application and aggregate size did not alter the Shannon diversity of diazotrophs but significantly changed the diazotrophic community structure. The variation of diazotrophic community structure explained by manure application was greater than that by aggregate size. Manure application promoted the relative abundance of Firmicutes but reduced that of α-Proteobacteria. Stochastic processes played a dominant role in the assembly of diazotrophs in the control treatment. Low-rate manure (9 Mg ha^−1^) application, rather than medium-rate (18 Mg ha^−1^) and high-rate (27 Mg ha^−1^) manure, significantly increased the relative importance of deterministic processes in diazotrophic community assembly. Taken together, our findings demonstrated that long-term manure application increased *nifH* gene abundance and altered the community structure and assembly process of diazotrophs in soil aggregates, which advanced our understanding of the ecophysiology and functionality of diazotrophs in acidic Ultisols.

## Introduction

The overuse of mineral N fertilizers in arablex soils has resulted in a series of environmental issues, such as water eutrophication, soil acidification, and nitrous oxide (N_2_O) emissions (Gallejones et al., 2012; Shcherbak et al., 2014), and accerelated the global climate change. Biological nitrogen (N) fixation, a process converting atmospheric dinitrogen (N_2_) into plant- or microorganism-available ammonium, is an important source of N input in ecosystems (Galloway et al., 2004). More importantly, biological N fixation is commonly recognized as an environmental-friendly approach alternative to fertilization to meet the N requirements of plants (Cleveland et al., 1999; Wang J. et al., 2020). Diazotrophs are the major biological engines for fixing atmospheric N into nitrogenous reactive compounds *via* the nitrogenase enzyme (Kuypers et al., 2018). The *nifH* gene, encoding nitrogenase, serves as an ideal biomarker to detect the abundance and community structure of diazotrophs without the need for cultivation (Reardon et al., 2014; Pajares and Bohannan, 2016; Tang et al., 2021).

Many biotic and abiotic variables can regulate the abundance and community structure of diazotrophs, including temperature (Fu et al., 2014), soil moisture (Feng et al., 2019), soil pH (Wang Y.et al., 2017), nutrient contents (Zheng et al., 2019), and agronomic practices (Xun et al., 2018). Fertilizer is an important abiotic factor affecting the diazotrophic communities, which has become a growing concern in the past decade (Berthrong et al., 2014; Liao et al., 2018; Chen H. et al., 2021). In general, the application of mineral fertilizer, especially N fertilizer, suppresses N fixation and diazotrophs, and alters diazotrophic community composition (Fan et al., 2019; Chen H. et al., 2021; Chen L. et al., 2021). However, the influence of organic fertilizers on diazotrophic abundance was controversial in previous findings. Chen H. et al. (2021) found that organic fertilizers application increased the abundance and activity of diazotrophs in a vertisol. Hu et al. (2018) demonstrated that manure application enhanced the abundance of *nifH* genes in a Mollisol. In contrast, manure amendment reduced the abundance of diazotrophs in an Ultisol through increasing soil pH (Lin et al., 2018a). To unveil the underlying mechanisms, it is imperative to investigate the effect of manure application on the abundance and community structure of diazotrophs in agroecosystems.

Soil has a complex hierarchical structure containing soil aggregates and pore space (Wilpiszeski et al., 2019). Different sized soil aggregates vary in their physicochemical properties, resulting in the variation of microbial community at the aggregate level (Trivedi et al., 2017; Han et al., 2018). Nutrients and labile C content are generally higher in macroaggregates and therefore select copiotrophic microbes, while higher recalcitrant C and lower nutrients content in microaggregates favor oligotrophic microorganisms (Trivedi et al., 2017). Bacterial community structures in aggregates have been well explored (Smith et al., 2014; Zheng et al., 2021), while less efforts have been devoted to study the impact of soil aggregation on the abundance and community structure of diazotrophs, especially under manure application conditions.

The community assembly mechanism of microorganisms is a central topic in microbial ecology (Kinnunen et al., 2018; Jiao et al., 2020). Although it is accepted that both deterministic and stochastic processes are important in microbial assembly, which process is more important remains largely unclear (Tripathi et al., 2018; Ning et al., 2020; Zhou et al., 2021). It has been shown that soil organic carbon and aggregate sizes could regulate the relative importance of different processes in the assembly of microorganisms (Dini-Andreote et al., 2015; Zheng et al., 2021). However, how manure application influences microbial community assembly remains largely unknown. Despite of some recent progress (Feng et al., 2018; Wang J. et al., 2020), we have limited knowledge of the relative contributions of deterministic and stochastic processes in diazotrophic community assembly, especially considering different sized soil aggregates in Ultisols.

The objectives of this study were (i) to explore the effect of pig manure application on the abundance, diversity, community structure and assembly process of diazotrophs in soil aggregates of Ultisols and (ii) to link the abundance of diazotrophs in soil aggregates to plant biomass and crop yield. We hypothesized that manure application would decrease abundance and alter community structure and assembly process of diazotrophs, due to the increased availability of N.

## Materials and methods

### Field experimental description and sampling

The experimental site was located at the Yingtan, Jiangxi Province, China (28°15′20″N, 116°55′30″E). This site has a typical subtropical monsoon climate, with an annual average precipitation of 1795 mm and temperature of 17.6°C. The tested soil is originated from the quaternary red clay and classified as an Ultisol. The long-term fertilization experiment began in 2002 and included four treatments with three replicates (12 plots in total): CK (without manure application and did not receive any type of fertilizer), M9 (receiving 9 Mg ha^−1^ of manure), M18 (receiving 18 Mg ha^−1^ of manure) and M27 (receiving 27 Mg ha^−1^ of manure). The manure application rate in M9 is approximately to the rate frequently used in local area. The experimental plots have received the designated manure applications yearly since 2002. The manure used in this study was pig manure collected from local farms and was stockpiled for 3 months. The characteristics of pig manure are shown in Supplementary Table 1. The cropping system is summer peanut followed by winter fallow. At harvest, plant biomass (belowground plus aboveground) and kernel samples were collected for analysis and oven dried at 65°C to a constant moisture level.

Soils were collected in October 2019 from a depth of 0–20 cm. Ten soil cores were collected at random from each plot and combined to form one soil sample. Soil samples were placed on ice and transported to the lab. Visible plant debris and stones were picked out and the remaining soils were passed through an 8 mm sieve and divided into two subsamples. One was used for aggregate fractionation, and the other was for soil variables determination. The methods for soil variables determination, such as pH, soil organic matter (SOM), total nitrogen (TN), ammonium, nitrate, and available phosphorus (AP) have been described previously by Lin et al. (2018b). Briefly, pH was determined by a glass electrode with a 1:5 soil-to-water ratio. SOM and TN were measured using the wet oxidation redox titration and micro-Kjeldahl methods, respectively. Ammonium and nitrate were analyzed by a continuous-flow analyzer. AP was measured using the molybdenum blue method.

### Soil aggregate fractionation

A wet-sieving technique was utilized for the fractionation of soil aggregate according to Elliott (1986) and Ye et al. (2021). Four fractions of soil aggregates for each sample were obtained: large macroaggregates (>2 mm), small macroaggregates (0.25–2 mm), microaggregates (0.053–0.25 mm), and silt and clay (<0.053 mm). An aliquot of each fraction was freeze-dried for DNA extraction.

### DNA extraction and quantitative PCR (qPCR)

DNA was extracted from 0.25 g freeze-dried aggregate soils using the MoBio PowerSoil™ DNA Isolation Kits (Qiagen, Carlsbad, CA) following the manufacturer's recommendations. The quality of the extracted DNA was evaluated using 1.2% agarose gel electrophoresis. The gene copy numbers of *nifH* were determined in a CFX96 Optical Real-Time Detection System as described by Lin et al. (2018a), using the primers nifHF/nifHR (Töwe et al., 2010). The amplification always resulted in a single peak with efficiencies of 92–95%, and R^2^ of 0.994–0.999.

### Sequencing and bioinformatics analysis

The primer pair used for high-throughput sequencing was the same as that for qPCR. The paired-end sequencing (2 × 300 bp) was conducted on an Illumina MiSeq platform by Majorbio Bio-Pharm Technology Co., Ltd. (Shanghai, China). After sequencing, the raw *nifH* reads were quality-filtered and merged using FLASH (version 1.2.7). The resulting reads were processed using QIIME v1.8.0 (Caporaso et al., 2010) to remove low quality sequences. After sorting sequences, barcode and primer sequences were eliminated. Chimeras and sequences with low similarity to the *nifH* sequence were subsequently discarded (Lin et al., 2018a), and the remaining high-quality sequences at a 95% similarity cutoff were assigned to the same OTUs using UPARSE (Edgar, 2013). Representative sequences from each OTU were taxonomically classified by constructing neighbor-joining phylogenetic trees in MEGA-X. The sequences were rarified before Shannon diversity calculation in QIIME. All sequences have been deposited in the DNA Data Bank of Japan under the accession number DRA014094.

### Data analysis

Statistical analyses were conducted using SPSS Statistics for Windows v 25.0 (IBM, Armonk, NY). The significance of difference in soil variables, plant biomass, peanut yield, the abundance of *nifH* genes among different treatments were evaluated by one-way analysis of variance (ANOVA) followed by least significant difference test. Pearson's correlation coefficients were used to determine the relationships between the abundance of *nifH* genes in aggregates and plant biomass or peanut yield. Non-metric multidimensional scaling (NMDS) analysis was performed using *metaMDS* function in the vegan package on the R platform (Version 4.1.0). Permutational multivariate analysis of variance (PERMANOVA) was conducted to determine the effects of manure applications and aggregate sizes on diazotrophic community structure using the *adonis* function in the vegan package. The relative importance of diazotrophic community assembly processes was evaluated by a phylogenetic bin-based null model framework, iCAMP (Ning et al., 2020). The analysis was conducted using the iCAMP (version 1.3.2) with appropriate default settings on a Galaxy platform (http://ieg3.rccc.ou.edu:8080). The significance of difference in the relative importance of specific ecological process was calculated based on bootstrapping with 1,000 replicates.

## Results

### Soil physicochemical variables, plant biomass and peanut yield

**Figure 1:**
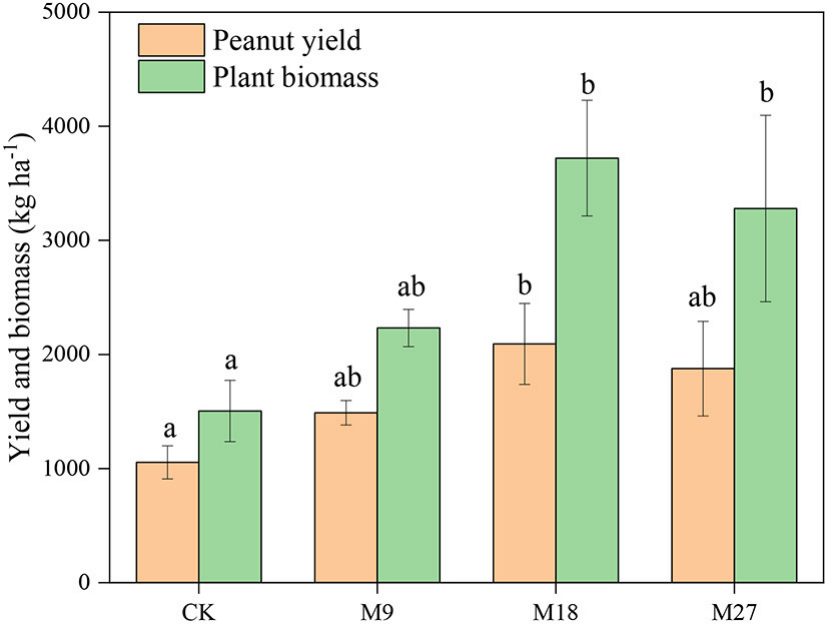
Peanut yield and plant biomass under long-term manure application. Vertical bars represent standard error (n = 3). Different letters denote significant differences (*p* < 0.05) between different treatments.

Compared to CK treatment, low-rate and medium-rate manure applications significantly decreased soil pH (Supplementary Table 2). However, the contents of SOM, TN, ${\text{NH}}_{4}^{+}$, ${\text{NO}}_{3}^{-}$ and AP increased with the application rate of manure, with the highest values recorded in the M27 treatment. The mass proportion of large macroaggregates was 1.76% in CK, and the value increased to 5.62 with high-rate manure applications (Supplementary Figure 1). Conversely, high-rate manure applications significantly decreased the mass proportion of microaggregates from 39.93% in CK to 34.27%. The plant biomass and peanut yield in CK was 1503 and 1054 kg ha^−1^, respectively. The application of manure generally increased the plant biomass and yield of peanut, with the highest values found in M18 (Figure 1).

### The abundance of *nifH* genes in soil aggregates and their correlation with plant biomass and yield

**Figure 2:**
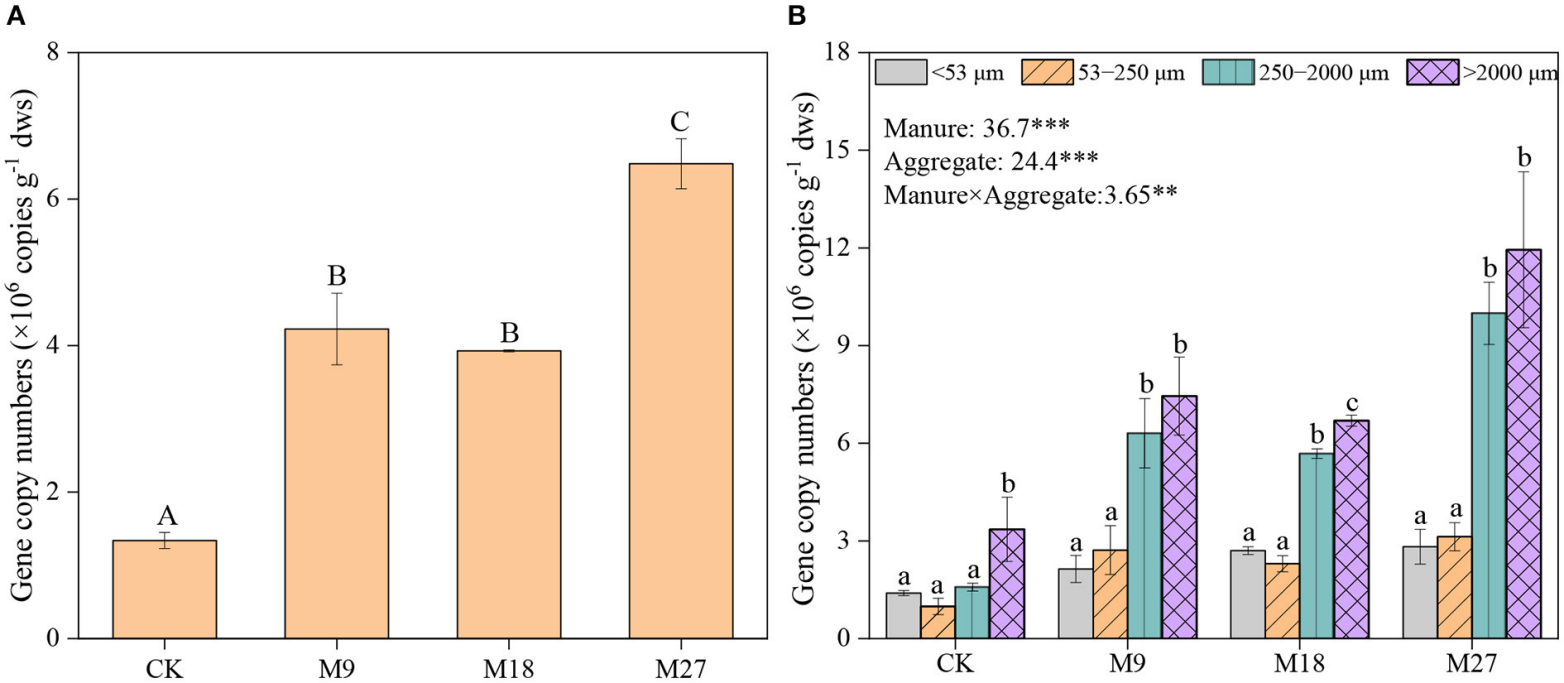
Abundance of *nifH* gene in soils **(A)** and aggregates **(B)** following long-term manure application. Vertical bars represent standard error (n = 3). Different lowercase letters denote significant differences (*p* < 0.05) between aggregate sizes in the same treatment, and different capital letters denote significant differences between different treatments. *** and ** indicate statistically significant at the 0.001 and 0.01 probability levels, respectively, while the numbers are *F* values by two-way ANOVA.

**Figure 3:**
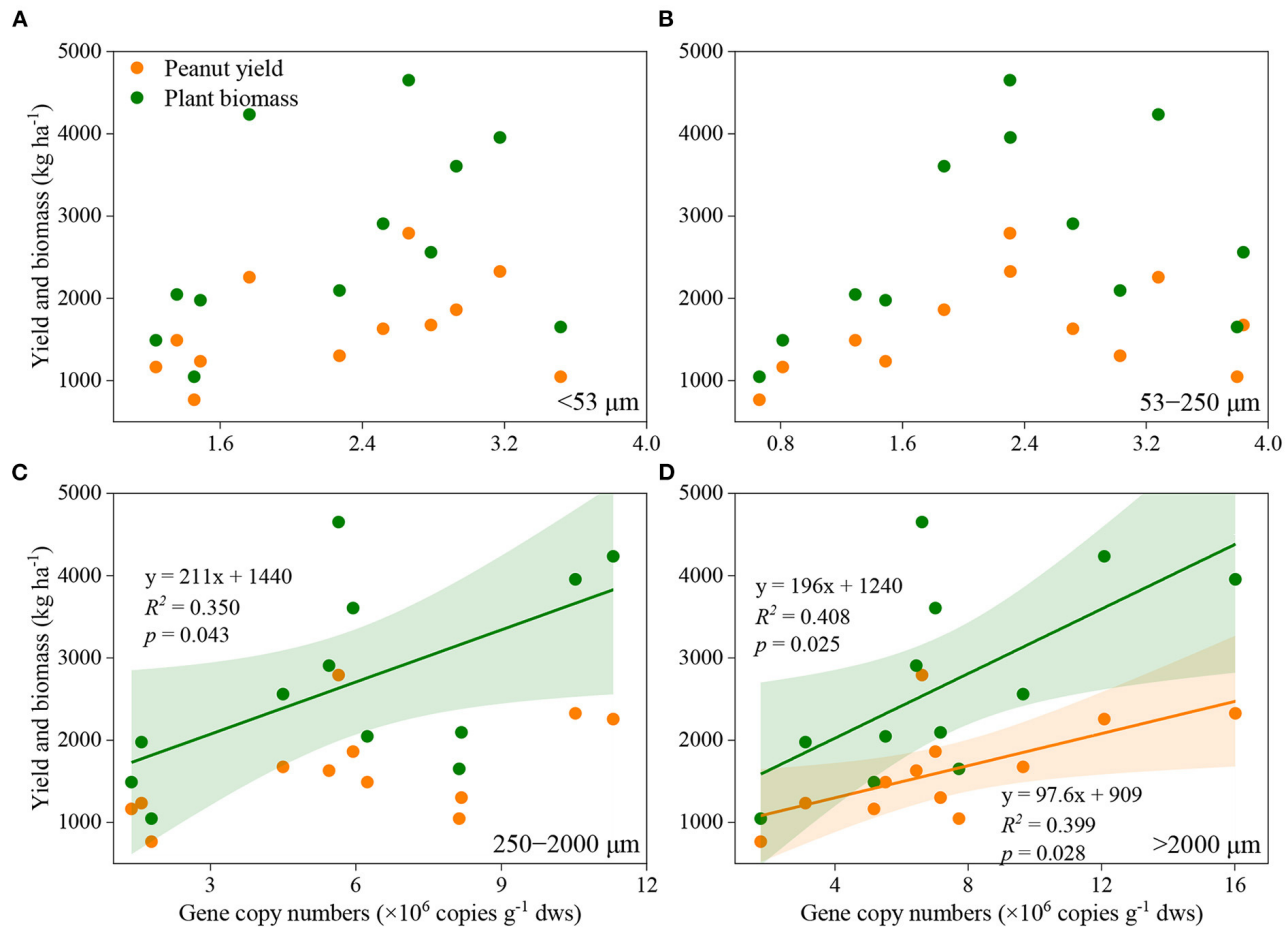
Association between peanut yield or plant biomass and the abundance of nifH gene in silt and clay **(A)**, microaggregates **(B)**, small macroaggregates **(C)**, and large mcroaggregates **(D)**. The shaded area indicates the 95% confidence interval of the regression models.

Manure application and aggregate size, and their interactions significantly influenced the abundance of *nifH* genes (Figure 2). In general, *nifH* gene abundance increased with the application rate of manure, regardless of aggregate sizes. The abundance of *nifH* genes was always higher in macroaggregates than in microaggregates and silt and clay. Pearson correlation analysis revealed that the abundance of *nifH* genes was significantly associated with plant biomass and peanut yield in large macroaggregates, and with plant biomass in small macroaggregates, but not in microaggregates and silt and clay (Figure 3; Supplementary Table 3).

### Diversity and community structure of diazotrophs

**Figure 4:**
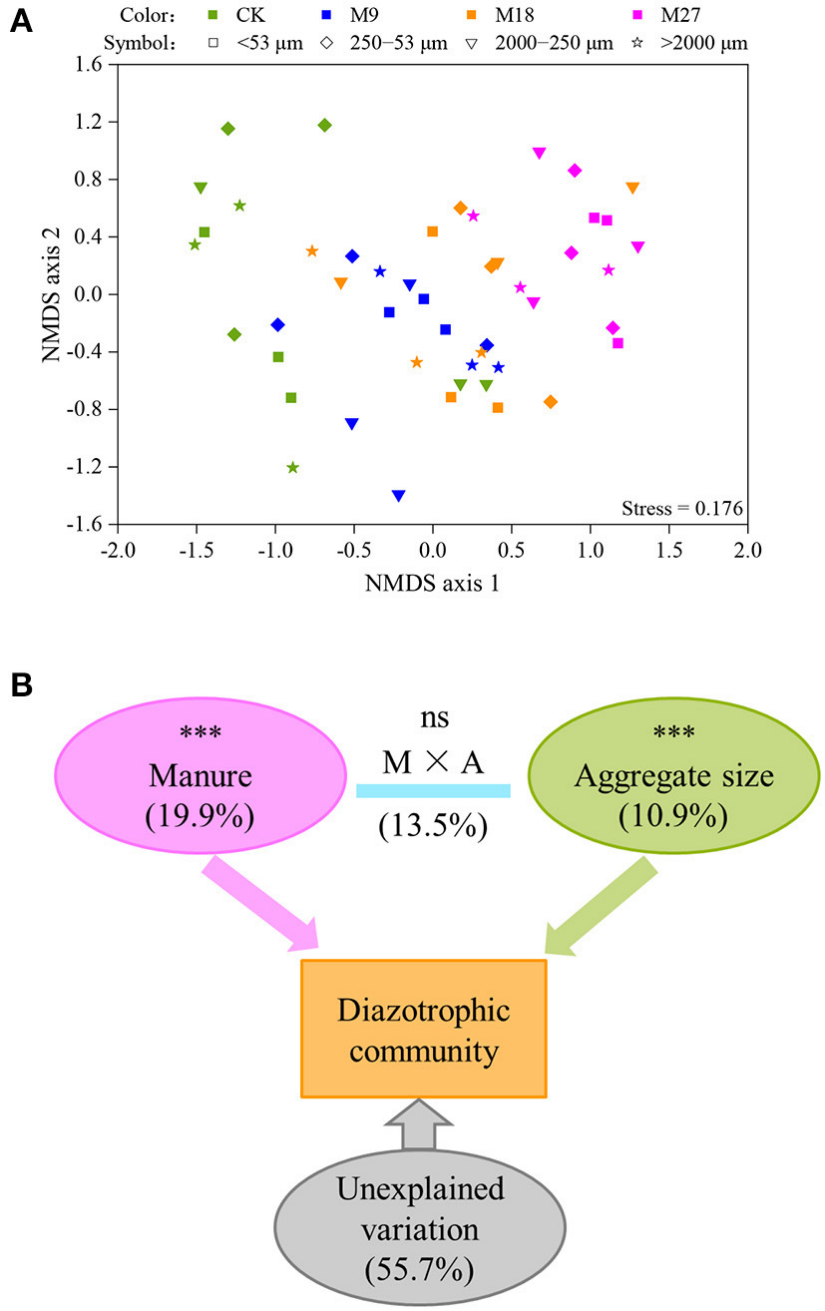
NMDS **(A)** and PERMANOVA **(B)** analysis of the communities of diazotrophs. Different colors represent treatments and different symbols indicate aggregate sizes in panel **(A)**. In panel **(B)**, the numbers are the percent of variation explained, and the letters M and A denote manure and aggregate sizes, respectively. Asterisks (***) denote significant differences at *p* < 0.001.

A total of 1,008,453 high-quality *nifH* sequences were obtained from 48 soil aggregate samples, with an average of 21,009 sequences per sample. Both manure application and aggregate sizes did not influence the Shannon diversity of diazotrophs (Supplementary Figure 2). However, the PERMANOVA analysis showed that manure application and soil aggregation significantly influenced the community structure of diazotrophs (Figure 4). The variation explained by manure application was 19.9%, which was higher than that explained by soil aggregate sizes (10.9%).

**Figure 5:**
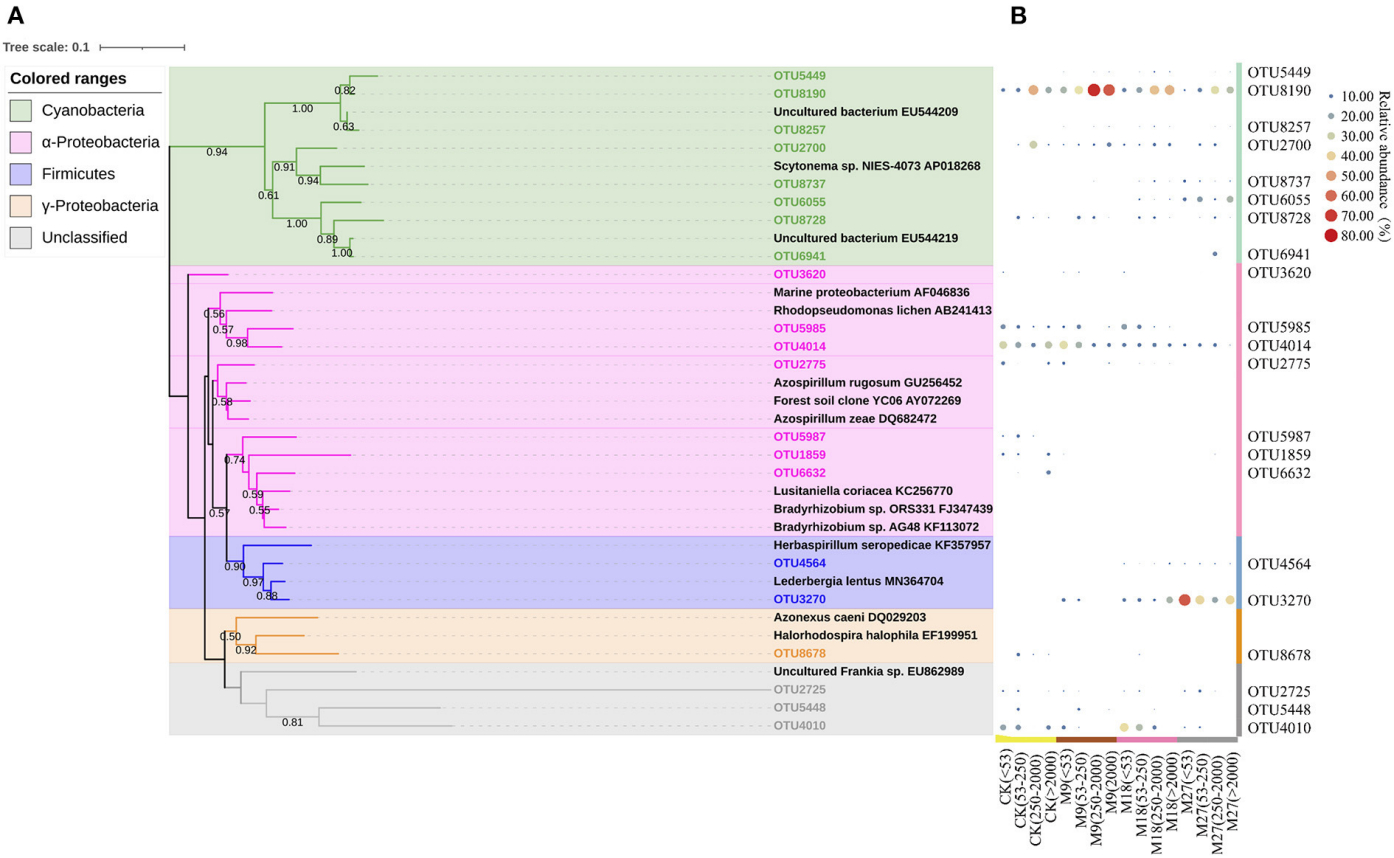
Phylogenetic tree shows the relationship between the representative sequences from this study and reference sequences from GenBank **(A)**. The colored ranges indicate different phylotypes of diazotrophs. Bootstrap values of > 50% based on 1,000 replicates are shown next to the branches. The scale bar represents 0.1 nucleic acid sequence divergence. Panel **(B)** shows the heatmap of the relative abundance of each OTU to the diazotrophic sequences in each treatment. The relative abundances of OTUs are indicated by bubble size.

**Figure 6:**
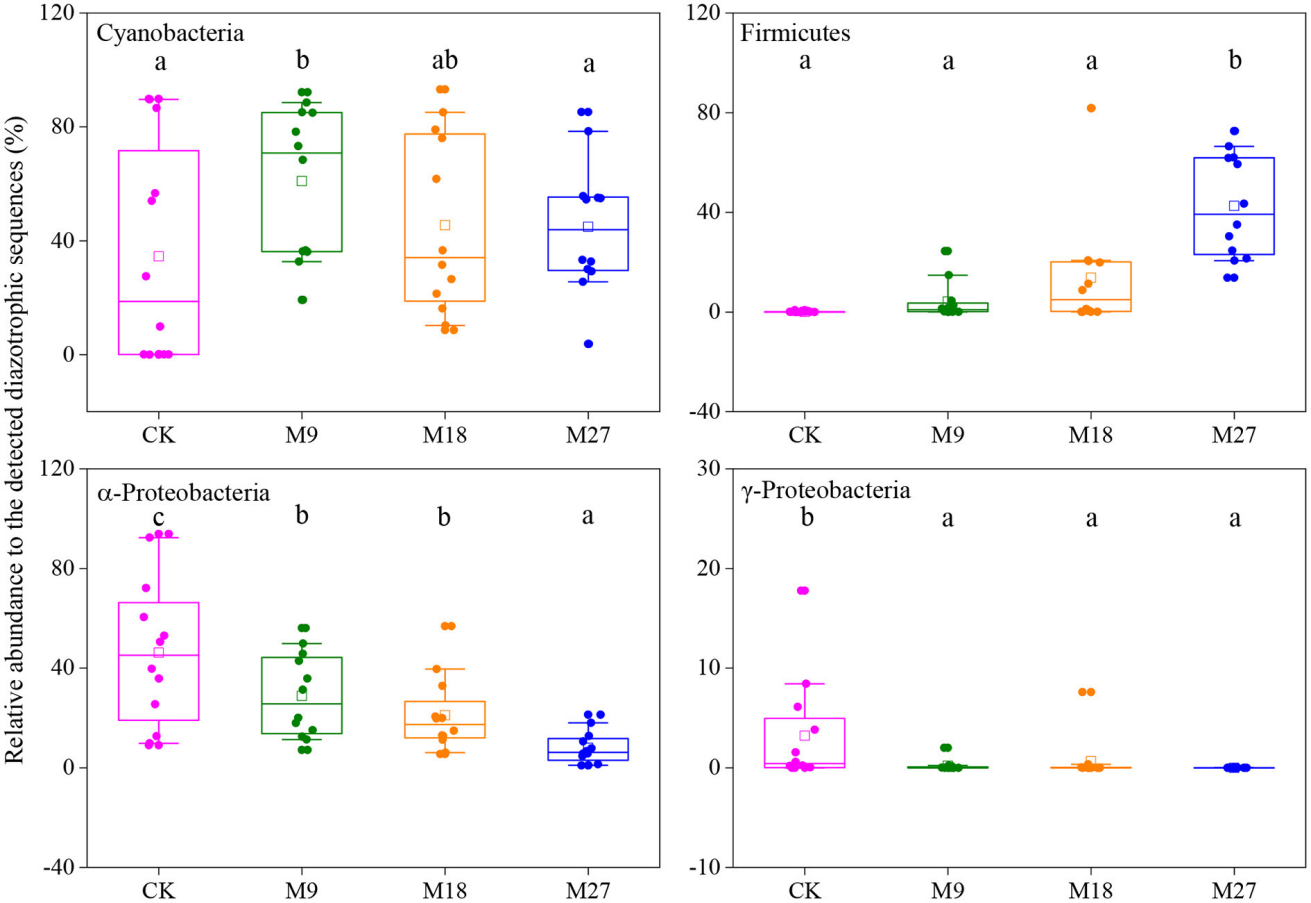
Box plots of main diazotrophic phyla affected by long-term manure applications. Boundaries of boxes indicate the first and third quartiles, and lines and squares within boxes represent the median and average, respectively. Whiskers indicate the 10th and 90th percentiles, and all the data are shown as dots. Different letters represent significant differences between treatments (*p* < 0.05).

Phylogenetic analysis showed that the sequences of *nifH* genes could be affiliated with four phylotypes, i.e., Cyanobacteria, α-Proteobacteria, γ-Proteobacteria and Firmicutes (Figure 5). Cyanobacteria was the dominant diazotrophs in the tested soils, accounting for 46.48% of the detected diazotrophic sequences (Figure 6). The application of low-rate manure, rather than medium-rate and high-rate manure, significantly increased the relative abundance of Cyanobacteria. The relative abundance of Firmicutes was 0.09% in the CK and increased to 42.62% after application of high-rate manure. The relative abundance of α-Proteobacteria and γ-Proteobacteria was the highest in the CK treatment and was reduced by the application of manure. Soil aggregate sizes did not significantly affect the relative abundances of Cyanobacteria, α-Proteobacteria, γ-Proteobacteria and Firmicutes (Supplementary Figure 3).

### Stochastic vs. deterministic diazotrophic community assembly

**Figure 7:**
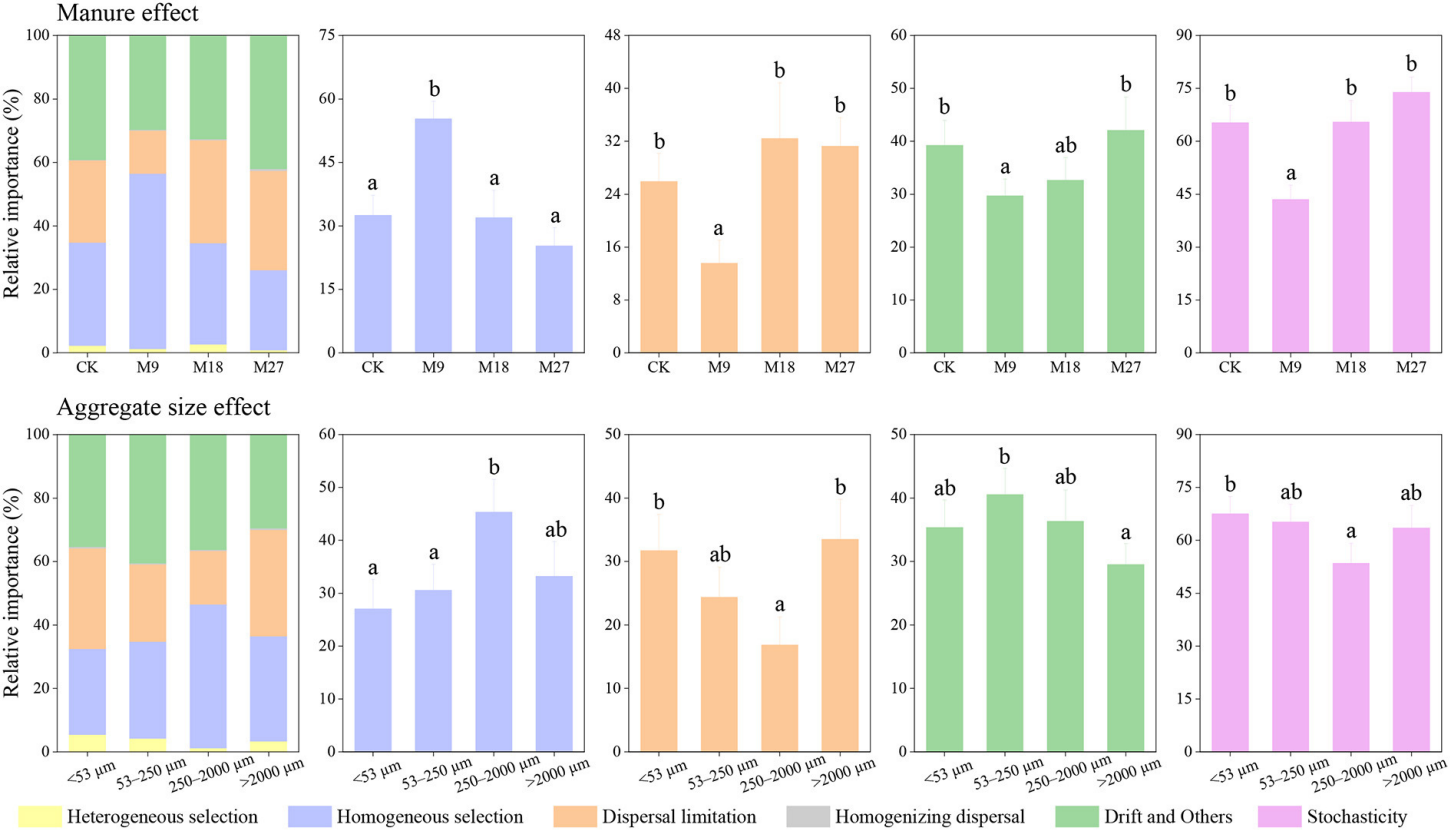
Relative importance of different ecological processes in diazotrophic community assembly based on iCAMP. Different letters denote significant differences (*p* < 0.05) based on bootstrapping with 1,000 replications. Stochasticity was estimated by the sum of dispersal limitation, homogenizing dispersal, and drift and others.

We investigated the community assembly mechanisms of diazotrophs using iCAMP. We found that homogenous selection, dispersal limitation, and drift and others were the main processes driving the community assembly of diazotrophs (Figure 7). Compared with CK, the application of low-rate manure significantly increased the relative importance of homogenous selection, while decreased the relative importance of dispersal limitation, and drift and others. The application of low-rate manure significantly decreased the relative contribution of stochastic process in diazotrophic community assembly. In addition, the relative importance of stochastic process in diazotrophic community assembly was also lower in the small macroaggregates than in other sizes of aggregates.

## Discussion

### Effect of manure and aggregate size on diazotrophic abundance in acidic Ultisols

Compared with NPK, the combined application of NPK and pig manure has been shown to reduce the abundance of *nifH* genes in an acidic Ultisol, possibly because of the promotion of soil pH (Lin et al., 2018a). Indeed, the diazotrophic communities in Ultisols have been acclimatized to acidic environments, and the increase of soil pH might be detrimental for them. However, this is not the case in this study, since we found that compared with control, the application of pig manure did not increase soil pH but increased the abundance of *nifH* genes. This result also rejected our hypothesis that the input of large amount of nutrients by manure applications would increase N availability and suppress the growth of diazotrophs. Several reasons may account for the increase of *nifH* genes by pig manure applications. Firstly, the majority of diazotrophs are heterotrophic or mixotrophic, and pig manure amendments provide C and energy sources for their growth (Rahav et al., 2016). The increase of SOC has been frequently found to stimulate the growth of diazotrophs (Perez et al., 2014; Liu et al., 2019), and the improvement of C substrate stimulates N fixation (Orr et al., 2012; Wang C. et al., 2017). Secondly, numerous studies have demonstrated the importance of phosphorus (P) availability in the growth of diazotrophs (Yang et al., 2019; Wang C. et al., 2020), since biological N fixation is energetically expensive and requires P for ATP synthesis (Olivares et al., 2013). However, Ultisols are well-known for low P availability, due to their strong P-fixation capacity by soil minerals. Thus, manure application could increase *nifH* gene abundance through substantially promoting the available P status. Thirdly, manure application increased the proportion of large macroaggregates, and the abundance of *nifH* genes increased with the increase of aggregate sizes. In general, SOC and nutrients in the macroaggregates are more abundant than those in the microaggregates (Trivedi et al., 2015; Ye et al., 2021), which could provide more C and energy sources for diazotrophs. Moreover, the cultivation of crops favored the growth of diazotrophs in larger aggregates, since microbes are more likely to be affected by plant roots in the macroaggregates than in the microaggregates (Zheng et al., 2018).

Besides biological N fixation, diazotrophs stimulate plant growth through diverse mechanisms, such as improving soil nutrient uptake efficiency, providing phytohormones to the host, and enhancing the host tolerance against stress (Dobbelaere et al., 2003; Shin et al., 2016; Pankievicz et al., 2021). As a result, the abundances of diazotrophs such as *Azospirillum* and *Burkholderia*, have been frequently found to be positively correlated with crop biomass and yield (Skonieski et al., 2019; Chen L. et al., 2021). Thus, it is reasonable to observe the close association between the abundance of *nifH* genes and plant performance. However, in this study, we found a significant correlation between the abundance of *nifH* genes in macroaggregates and plant biomass, rather than in microaggregates, indicating that the diazotrophs in macroaggregates more likely contribute to plant performance. As a result, the abundance of *nifH* genes in large macroaggregates might be a good indicator for plant performance in acidic Ultisols. It should be noted that only DNA was retrieved and analyzed in this study, and the higher abundance of *nifH* genes does not necessarily mean the higher activity of nitrogen fixation. Further investigations of the *nifH* transcription and nitrogen fixation activity are required in the future studies.

### Effect of manure and aggregate size on diazotrophic community structure in acidic Ultisols

Pig manure application significantly altered the diazotrophic community structure in this study. Previous studies have shown that the application of mineral or organic fertilizers introduced a large amount of nutrients and C sources into soils, which significantly influenced the community structure of diazotrophs (Hu et al., 2018; Liao et al., 2018; Chen H. et al., 2021). Shi et al. (2021) further showed that organic manure over mineral fertilization primarily determined the stability of diazotrophic community structure in an upland chromic cambisol. Moreover, aggregate size also significantly influenced the community structure of diazotrophs, which was in line with most of previous studies that aggregate size classes substantially affected soil microbial community structure (Lin et al., 2019; Wang et al., 2021). Indeed, various sizes of aggregates could provide heterogeneous niches for diazotrophs and selected different diazotrophic phylotypes. However, pig manure was more important than aggregate sizes in regulating the community structure of diazotrophs, indicating the diazotrophic communities in acidic Ultisols are more prone to be influenced by resource availability than by the sizes of niches.

Cyanobacteria have been frequently reported to be dominant diazotrophs in aquatic environments (Liao et al., 2018; Wang et al., 2019). Cyanobacteria were also found to be dominant in the tested soils, indicating that the photoautotrophic diazotrophic species could also thrive in upland soils with high temperature and precipitation, due to their ability to endure repeated desiccation and hydration (Teng et al., 2009; Tang et al., 2021). Li et al. (2018) showed that Cyanobacteria may require more N than other diazotrophs, and thus the application of low-rate manure increased the relative abundance of Cyanobacteria through providing more nutrients. However, this effect might be counteracted by the addition of large amount of organic C in the medium-rate and high-rate manure treatments, since the autotrophic Cyanobacteria were less competitive than heterotrophic diazotrophs when large amount of organic C was applied. Moreover, the application of pig manure, especially in the high-rate, increased the relative abundance of Firmicutes. Firmicutes are generally the most abundant bacterial phylum in manure, and are able to degrade the complex organic compounds and thrive in soils after fertilization (Rieke et al., 2018; Ye et al., 2022). Numerous studies have shown that the application of manure increased the relative abundance of Firmicutes in soils (Francioli et al., 2016; Xun et al., 2016; Ye et al., 2019). In contrast, manure application reduced the relative abundance of α-Proteobacteria. This is out of our expectation since α-Proteobacteria are generally regarded as copiotrophic microorganisms (Eilers et al., 2010; Hu et al., 2018). It is likely that the α-Proteobacteria diazotrophs were less competitive than diazotrophic Firmicutes after pig manure applications and were out-competed in the manure-amended treatments, due to the overwhelming dominant role of Firmicutes in these treatments. Moreover, different OTUs within a diazotrophic groups may respond differently to manure applications (Figure 5B), indicating the highly diverse physio-ecological characteristics of diazotrophs in acidic Ultisols.

### Effect of manure and aggregate size on diazotrophic community assembly in acidic Ultisols

We found that stochastic processes played a dominant role in the assembly of diazotrophs in CK. However, the application of low-rate manure, but not medium-rate and high-rate manure, significantly increased the relative importance of deterministic processes in diazotrophic community assembly. The possible explanation is that low-rate manure reduced soil pH in the tested soils. Soil pH was generally regarded as an important factor driving the assembly of diazotrophs (Wang Y.et al., 2017), and the lower pH in acidic soils would aggravate the stress and strengthen environmental filtering (Feng et al., 2018). As a result, the application of low-rate manure reduced soil pH, which further caused an increasing dominance of deterministic processes in diazotrophic community assembly. Although medium-rate manure also decreased soil pH, it increased the nutrient content to a greater extent. A previous study has shown that the relative importance of stochastic processes in microbial community assembly increased with the addition of nutrients (Zhou et al., 2014), since the elevation of nutrient content was beneficial for microbial growth, which would stimulate the stochastic processes (Wang J. et al., 2020). Thus, the increasing deterministic processes by reducing soil pH was counteracted by the increasing stochastic processes by promoting nutrient content in the medium-rate manure treatment. The variation of soil pH and nutrient content after fertilization might play a crucial role in determining the assembly processes of diazotrophs (Wang J. et al., 2020). Moreover, we found that the relative importance of stochastic processes in diazotrophic community assembly was the lowest in the 250–2000 μm aggregates, indicating that aggregate sizes could also play an important role in regulating the assembly of diazotrophic communities. However, the underlying mechanisms require further investigations.

## Conclusions

Our study provided novel evidence that both manure application and soil aggregation increased the abundance of *nifH* genes. The abundance of *nifH* genes in large macroaggregates was positively associated with plant biomass and peanut yield, which was not the case in microaggregates and silt and clay. Manure applications had a greater role than aggregate sizes in regulating the community structure of diazotrophs. Manure applications increased the relative abundance of Firmicutes while reduced that of α-Proteobacteria. Moreover, the assembly of diazotrophs in CK was mainly dominated by stochastic processes, and low-rate manure application increased the relative importance of deterministic processes in diazotrophic community assembly. Together, our study suggested that manure application could substantially influence the abundance, community structure and assembly process of diazotrophs in aggregates of acidic Ultisols.

## Data availability statement

The datasets presented in this study can be found in online repositories. The names of the repository/repositories and accession number(s) can be found in the article/Supplementary material.

## Author contributions

YL designed the research and draft the manuscript. GY and JF conducted the experiment and performed the data analysis. H-WH revised the manuscript. J-ZH supervised all aspects of experimentation and manuscript preparation. All authors contributed to the final version.

## Funding

This work was financially supported by the National Natural Science Foundation of China (42077041 and 41930756).

## Conflict of interest

The authors declare that the research was conducted in the absence of any commercial or financial relationships that could be construed as a potential conflict of interest.

## Publisher's note

All claims expressed in this article are solely those of the authors and do not necessarily represent those of their affiliated organizations, or those of the publisher, the editors and the reviewers. Any product that may be evaluated in this article, or claim that may be made by its manufacturer, is not guaranteed or endorsed by the publisher.
