# Supplementary material for: Manure applications alter the abundance, community structure and assembly process of diazotrophs in an acidic Ultisol
Source: Front Microbiol. 2022 Aug 12;13:965293. doi: 10.3389/fmicb.2022.965293 (PMC9412762; doi:10.3389/fmicb.2022.965293)
Supplement: Supplementary file 1 [file Data_Sheet_1.docx]

Supplementary Material

# Supplementary Figures and Tables

## Supplementary Figures

##

**Supplementary Figure 1.** Mass proportion of aggregates following long-term application of different rates of pig manure.

##

**Supplementary Figure 2.** Shannon diversity of diazotrophs in different soil aggregates following manure applications. Vertical bars represent standard error of the mean (SEM; n = 3).

**Supplementary Figure 3.** Box plots of main diazotrophic phyla affected by soil aggregate sizes. Boundaries of boxes indicate the first and third quartiles, and lines and squares within boxes represent the median and average, respectively. Whiskers indicate the 10th and 90th percentiles, and outliers are shown as dots. Different letters represent significant differences between treatments (P < 0.05).

## Supplementary Tables

**Supplementary table 1** Properties of pig manure.

| Organic material | Organic C  (g C kg^−1^) | Total N  (g N kg^−1^) | Total P  (g P kg^−1^) | Total K  (g K kg^−1^) | Recalcitrance index |
| --- | --- | --- | --- | --- | --- |
| Pig manure | 157 | 20.1 | 12.1 | 7.9 | 0.47 |

Values are presented as means (n = 3). Recalcitrance index were determined using solid-state ^13^C nuclear magnetic resonance spectroscopy.

**Supplementary table 2** Soil physicochemical properties following long-term application of different rates of pig manure.

| Treatments | pH  (1:5 H_2_O) | SOM  (g C kg^–1^) | TN  (g N kg^–1^) | NH_4_^+^  (mg N kg^–1^) | NO_3_^–^  (mg N kg^–1^) | AP  (mg P kg^–1^) |
| --- | --- | --- | --- | --- | --- | --- |
| CK | 4.95b | 5.68a | 0.66a | 7.52a | 5.95a | 6.39a |
| M9 | 4.80a | 6.61ab | 0.81ab | 8.31ab | 8.85b | 28.53ab |
| M18 | 4.79a | 6.73bc | 0.81b | 9.41b | 11.17c | 49.75b |
| M27 | 4.90ab | 7.67c | 0.90b | 9.62b | 11.80c | 104.95c |

Values within the same column followed by different letters indicate significant differences at *p* <0.05.

CK, no manure; M9, low-rate manure with 9 Mg ha^–1^ y^–1^; M18, medium-rate manure with 18 Mg ha^–1^ y^–1^; M27, high-rate manure with 27 Mg ha^–1^ y^–1^; SOM, soil organic matter; TN, soil total nitrogen; AP, available phosphorus.

**Supplementary table 3** Results of Pearson correlation analysis between peanut yield or plant biomass and the abundance of *nifH* gene in different sizes of aggregates.

| Aggregate  size | Peanut yield | | |  | Plant biomass | | |
| --- | --- | --- | --- | --- | --- | --- | --- |
|  | *R* | *p* | Equation |  | *R* | *p* | Equation |
| <53 μm | 0.361 | 0.249 | - |  | 0.410 | 0.185 | - |
| 53−250 μm | 0.290 | 0.361 | - |  | 0.337 | 0.284 | - |
| 250−2000 μm | 0.537 | 0.072 | - |  | 0.591 | 0.043 | y = 211x + 1440 |
| >2000 μm | 0.631 | 0.028 | y = 97.6x + 909 |  | 0.639 | 0.025 | y = 196x + 1240 |
